# A cross-sectional survey to assess the risk factors associated with stigmatizing attitudes towards patients with podoconiosis among rural youth in southern Ethiopia

**DOI:** 10.1093/trstmh/traa091

**Published:** 2020-11-09

**Authors:** Kibur Engdawork, Gail Davey, Desta Ayode, Colleen M McBride, Getnet Tadele

**Affiliations:** Department of Sociology, College of Social Sciences, Addis Ababa University, Addis Ababa, 1176, Ethiopia; Brighton and Sussex Medical School, University of Sussex, Falmer, Brighton BN1 9PX, UK; Department of Sociology, College of Social Sciences, Addis Ababa University, Addis Ababa, 1176, Ethiopia; Rollins School of Public Health, Emory University, Atlanta, GA 30322, USA; Department of Sociology, College of Social Sciences, Addis Ababa University, Addis Ababa, 1176, Ethiopia

**Keywords:** Ethiopia, gene–environment, podoconiosis, risk factors, stigmatizing attitudes, youth

## Abstract

**Background:**

Many health conditions are associated with stigma due to beliefs about their causes and the physical changes experienced by patients. Among such conditions are several neglected tropical diseases (NTDs). Podoconiosis, classified as an NTD, is a form of lymphoedema caused by the co-influence of genetic and environmental factors. It is a major public health problem in Ethiopia and is associated with intense stigma. Despite this, little is known about the factors contributing to stigmatizing attitudes against patients with the disease.

**Methods:**

A cross-sectional survey was conducted in southern Ethiopia to analyse the attitudes of rural youth and associated risk factors for stigmatizing attitudes towards patients with podoconiosis, with the aim of informing stigma reduction strategies.

**Results:**

The survey included 336 randomly selected youth. Of the 177 (52.7%) youth who held more stigmatizing attitudes toward patients with podoconiosis, 105 (59.3%) were females and 171 (96.6%) did not have affected friends. Accurate knowledge about gene–environment influences and rejection of infectious causes of podoconiosis were associated with less stigmatizing attitudes.

**Conclusions:**

Improving understanding of the gene–environment interaction and dispelling beliefs about infectious causes may reduce negative attitudes about podoconiosis. Affected youth may play an important role as agents of change to spread non-stigmatizing messages.

## Introduction

Stigma has been a topic of growing interest across many disciplines due to its dehumanizing effect on people who experience it. Researchers are focussing much attention on public stigma and stigmatizing attitudes. The tendency for people to recognize patients not as whole individuals, but as people with particular socially undesirable characteristics, is noteworthy.^1^

Erving Goffman's pioneering work on stigma has inspired a number of studies, particularly in the field of social sciences.^2^ Goffman conceptualized stigma as an attribute that is deeply discrediting with the potential to spoil the social identity of the bearer.^3^ Health-related stigma has been defined by Weiss and Ramakrishna as a ‘social process or related personal experience characterized by exclusion, rejection, blame, or devaluation that results from the experience of a person or group identified with a particular health condition’.^4^ Recent research advances promote cross-cutting approaches to investigate the individual and social domains of the stigmatization process and the intersectionality of other types of stigma with health-related stigma.^5,6^ The Health Stigma and Discrimination Framework, for example, posits that stigma intersects with other axes of disempowerment and marginalization, such as race, sexual orientation, class and gender, in ways that result in some people being more disadvantaged by health-related stigma.^5^ Thus responses to people with a particular condition may differ based on location and on local differences in social determinants of stigma, such as the beliefs, culture and socio-economic status of the individuals experiencing stigma or those perpetuating stigma.^2,6,7^ As a result, the labelling of patients is not always medically warranted with respect to the nature of the health problem itself.^4^ Not all health problems elicit uniformly negative reactions from others. Some people have more while others have less stigmatizing attitudes, and some people are exaggeratedly nice toward people with health conditions.^7^

Neglected tropical diseases (NTDs), conditions that are mainly caused by poverty and inequality, are often highly stigmatized. Previous research has shown significant stigma towards individuals affected by several NTDs, either due to fear of contracting the disease or the physical disfigurements that accompany them.^8–10^ Studies have also underlined how stigma resulting from specific NTDs contributes to disease burden and poverty.^8–10^

Podoconiosis is classified as an NTD.^11^ It is a non-filarial form of tropical lymphoedema resulting in bilateral swelling of the lower legs that develops when genetically susceptible individuals are exposed to irritant particles in volcanic soil when walking barefoot. Sensitivity to environmental risk factors for the disease are inherited rather than the disease itself being inherited.^12^ Approximately 4 million people are affected worldwide^13^ and there is current evidence of the presence of podoconiosis in 12 African countries.^14^ In Africa, Ethiopia carries the highest burden of podoconiosis with approximately 1.6 million patients.^15^

Podoconiosis elicits a high degree of stigma—ranging from disapproving reactions to negative sanctions^16,17^—that presents a major barrier to control and treatment of the disease.^17^ Stigma related to podoconiosis often contributes to poor health outcomes and life opportunities for the patient. Affected individuals are avoided by peers, neighbours and the wider community and are excluded from religious and educational settings. Despite stigma reduction efforts, the stigma experienced by podoconiosis patients and their families continues to negatively impact patients’ health and well-being.^18^

While the degree and consequences of stigma for patients with podoconiosis have been documented, less is known about risk factors for stigmatizing attitudes. Prior reports suggest that misconceptions among adults (e.g. belief in an infectious cause and heritability of podoconiosis) contribute to the intensification of social stigma against affected individuals,^18,19^ while accurate understanding lessens stigmatizing attitudes.^20^ However, there have been few attempts to document this quantitatively or to understand risk factors in groups other than adults (e.g. youth ages 15–24 y). In contrast, many studies have explored risk factors for stigmatizing attitudes towards patients with mental illness^21,22^ and people living with human immunodeficiency virus (HIV)/acquired immunodeficiency syndrome (AIDS) (PLWHA).^23,24^ Insufficient knowledge of disease aetiology is associated with stigmatizing attitudes towards people with mental illness^21^ and PLWHA^23,24^ and lower exposure to PLWHA is reported to intensify HIV-related stigma^25^ Conversely, many studies have demonstrated the significant contribution that accurate knowledge can make to reducing stigmatizing attitudes towards PLWHA.^23,24^

Assessing the impact of knowledge about disease in minimizing stigma is a priority in health-related stigma research.^4^ In order to identify ways to reduce stigma towards podoconiosis patients, it is crucial to understand factors that contribute to this stigma. The aim of the present study was to measure stigmatizing attitudes and associated risk factors among rural youth in southern Ethiopia using a cross-sectional survey.

## Materials and methods

### Study setting

A cross-sectional survey was conducted in two *kebeles* (lowest rural administrative units) of Wolaita Zone, southern Ethiopia. The Tome Gerira and Sura Koyo *kebeles* were selected because neither had been targeted in previous studies of podoconiosis.^19,20,26^

Wolaita Zone is an area of high prevalence of podoconiosis: >5% of the population is affected and many people are at risk through exposure to irritant soil.^27^ The zone is located in the Southern Nations, Nationalities and People's Regional State (SNNPRS). Based on a statistical abstract prepared by the Bureau of Finance and Economic Development (BoFED) in 2016, Wolaita Zone had a total population of 1 969 196, of whom 49.3% were males and 50.7% females.^28^

### Study design

This study is part of a larger project on enhancing Ethiopian youth's literacy on gene–environment contributions to health using the context of podoconiosis.^29^ Data were collected between August and September 2016. In line with the United Nations definition,^30^ youth ages 15–24 y were targeted. With the help of *kebele* officials, a census of youth in this age group in the participating *kebeles* was conducted prior to initiating the research. About 3542 youth were identified (1841 females and 1701 males), of whom 383 were affected by podoconiosis. The optimal sample size needed to identify differences in responses of ±5 units with a 95% confidence interval (CI) was 347.^31^ Thus 377 youth were randomly selected from the census sampling frame in anticipation of 5% refusal to participate. As this study was designed to investigate public stigma towards patients with podoconiosis, 41 affected individuals were excluded and the remaining 336 unaffected youth were included in the analysis.

### Instrument and data collection

The questionnaire was initially developed in English, translated into the Amharic and Wolayitigna languages before being checked for accuracy and back-translated into English. Ten data collectors were recruited from the town of Wolaita Sodo using informal networks and received 3 d of training on the objectives of the study, the items included in the survey questionnaire and how to carry out the survey. The survey was pilot tested in two nearby rural *kebeles* (Sodo Zuria and Damot Woydie) with 30 youth. These youth did not take part in the main survey.

Data collectors used a paper questionnaire to interview youth in and around their homes in private locations. The interviews took 45–55 min. Written informed consent was obtained from all participants or from parents or guardians of those who were <18 y of age. The respondents were given exercise books and pens in recompense for their time. The study received ethical approval by the Institutional Review Board of the College of Health Science, Addis Ababa University, Addis Ababa, Ethiopia.

### Measures

The dependent variable, stigmatizing attitudes, was measured using six indicators (e.g., Are you willing to marry someone from a podoconiosis-affected family?), where the subject's response was assessed using a 3-point scale (1, definitely yes; 2, maybe; 3, definitely no). The indicators were taken from a prior study that adapted a social distance measure for the context of podoconiosis in Ethiopia.^20^ The scale showed good consistency (Cronbach's α=0.896). However, the Shapiro–Wilk test showed that scores for stigmatizing attitude were not normally distributed across the population (W=0.84, p<0.001). Thus the data were collapsed into two stigma categories using the median as a cut point. Median scores between 6 and 11 were considered ‘less stigmatizing attitudes’ and a median score ≥12 points was defined as having ‘more stigmatizing attitudes’ towards patients with podoconiosis.

The independent variables included sociodemographic characteristics such as gender, age, education level, rural youth's gene–environment knowledge and rejection of infectious causes, contact with health extension workers (HEWs) and friendship with patients with podoconiosis.

Gene–environment knowledge was based on four questions and adapted to the causal factors of podoconiosis.^13,32^ Youth were asked to rate each statement as ‘true’, ‘false’ or ‘I don't know’ (e.g. ‘Individuals are born with susceptibility to podoconiosis that gets triggered by walking barefoot’). Each accurate response about the influence of gene–environment on podoconiosis was assigned 1 point.

Rejection of infectious causes was assessed using 11 questions derived from prior research conducted in Ethiopia.^20^ Each response that rejected an infectious cause for podoconiosis was assigned 1 point.

Our previous report showed that youth who have contact with HEWs have more accurate knowledge of podoconiosis.^29^ Thus contact with HEWs, women trained in health promotion and disease prevention for ≥1 y and deployed in their local community, was assessed. Youth who had had contact with HEWs at home in the year prior to the survey were compared with those reporting no contact. Finally, friendship with patients with podoconiosis was assessed, as this may create opportunities for unaffected individuals to better understand the disease.

### Data analysis

The survey data were analysed using Stata version 13.0 (StataCorp, College Station, TX, USA). Frequencies and distributions were examined to check for out-of-range values and other errors in the data. After data cleaning, descriptive analyses were performed. Bivariate correlations were conducted to assess the relationship between youth characteristics and stigmatizing attitudes towards patients with podoconiosis. Cross-tabulation and χ^2^ tests were performed to check for statistical associations between groups based on stigmatizing attitudes. The level of statistical significance was set at p<0.001 (two-tailed). Variables that were found to be significantly associated during cross-tabulation were entered into a binary logistic regression model to examine their predictive ability. To control for potential confounding effects, the relationship between independent variables was assessed and the probability percentage was measured to assess the likelihood of having less (0) or more (1) stigmatizing attitudes by gene–environment knowledge and rejection of infectious causes.

## Results

### Study population

All 336 sampled youth agreed to take part in the study, of whom 51.5% were females and 71.1% were 15–18 y of age (Table 1). About 5% of the youth had no formal education, while most (71.1%) were reported as having a grade 7–12 education. A minority (7.1%) reported having friendships with affected individuals and 78.6% reported having had contact with HEWs at least once in the year prior to the survey.

**Table 1.** Demographics and characteristics of youth participating in the survey (N=336)

Characteristics	n (%)
Age (years)	
15–18	239 (71.1)
19–24	97 (28.9)
Gender	
Female	173 (51.5)
Male	163 (48.5)
Education level	
No formal education	16 (4.8)
Grades 1–6	69 (20.5)
Grades 7–12	239 (71.1)
Vocational/technical education	5 (1.5)
College/university	7 (2.1)
Has friendship with affected person	24 (7.1)
Had contact with a HEW in the past 12 months	264 (78.6)

### Stigmatizing attitudes towards patients with podoconiosis

Youth reported low willingness to marry someone from an affected family (mean 2.6 [standard deviation {SD} 0.7]), to help an affected person with treating their feet (mean 2.2 [SD 0.9]) or to make friends with an affected person (mean 2.1 [SD 1.0]) (Table 2).

**Table 2.** Percentage of willingness and unwillingness to have interpersonal interaction with affected individuals

Indicators of stigmatizing attitudes		n (%)
Willingness to live next door to affected person	Definitely yes	216 (64.3)
	Maybe	30 (8.9)
	Definitely no	90 (26.8)
Willingness to share meals with affected person	Definitely yes	204 (60.7)
	Maybe	23 (6.8%)
	Definitely no	109 (32.4)
Willingness to make friends with affected person	Definitely yes	145(43.2)
	Maybe	28 (8.3)
	Definitely no	163 (48.5)
Willingness to work with affected person on household chores	Definitely yes	167 (49.7)
	Maybe	30 (8.9)
	Definitely no	139 (41.4)
Willingness to help affected person with treating feet	Definitely yes	122 (36.3)
	Maybe	30 (8.9)
	Definitely no	184 (54.8)
Willingness to marry someone from affected family	Definitely yes	52 (15.5)
	Maybe	25 (7.4)
	Definitely no	259 (77.1)

### Factors associated with stigmatizing attitudes against patients with podoconiosis

Overall, 52.7% of the youth were found to have more stigmatizing attitudes towards patients with podoconiosis (Table 3). There was no significant association between age, level of education or contact with HEWs and stigmatizing attitudes. However, there was a significant positive correlation between female gender and more stigmatizing attitudes, with 59.3% of females demonstrating more stigmatizing attitudes compared with 40.7% of males. Conversely, having affected individuals as friends was significantly correlated with less stigmatizing attitudes.

**Table 3.** Association between demographic and social characteristics of youth participating in the survey and their stigmatizing attitudes toward patients with podoconiosis (N=336)

	Less stigmatizing attitudes,	More stigmatizing attitudes,	
Independent variable	n (%) (N=159)	n (%) (N=177)	p-Value
Age (years)			NS
15–18	107 (67.3)	132 (74.6)	
19–24	52 (32.7)	45 (25.4)	
Gender			<0.001
Female	68 (42.8)	105 (59.3)	
Male	91 (57.2)	72 (40.7)	
Level of education			NS
No formal education	7 (4.4)	9 (5.1)	
Formal education	152 (95.6%)	168 (94.9%)	
Friendship with patients			<0.001
Yes	18 (11.3)	6 (3.4)	
No	141 (88.7)	171 (96.6)	
Had contact with a HEW in the past 12 months			NS
Yes	123 (77.4)	141 (79.7)	
No	36 (22.6)	36 (20.3)	

Bivariate correlations were used to assess the relationship between youth characteristics and stigmatizing attitudes towards patients. Cross-tabulation and χ^2^ tests were performed for associated probabilities and to check for statistical associations between groups based on stigmatizing attitudes. The level of statistical significance was set at p<0.001 (two-tailed).

Percentages may not add up to 100% due to rounding.

NS: not statistically significant.

Summary statistics of variables used in the binary logistic regression model are shown in Table 4.

**Table 4.** Summary statistics of variables used in the logistic regression model

Variable	Mean	Minimum	Maximum	Label
Dependent				
Stigma level	0.52	0	1	0, less stigmatizing attitudes; 1, more stigmatizing attitudes
Independent				
Friendship with affected individuals	0.08	0	1	0, no; 1, yes
Gender	0.12	0	1	0, male; 1, female
Gene–environment knowledge	2.40	0	4	Number of accurate responses about gene–environment influences
Rejecting infectious causes	4.73	0	11	Accuracy in rejecting infectious causes

Male sex, friendship with affected people, accurate knowledge of gene–environment influences and rejection of infectious causes were all significantly associated with less stigmatizing attitudes towards patients (Table 5). Youth who had established friendships with patients had 0.23 (95% CI 0.08 to 0.75) times the odds of holding stigmatizing attitudes than those without. Females had 1.11 (95% CI 1.11 to 2.93) times the odds of having stigmatizing attitudes compared with males. For a 1-unit increase in rejection of infectious causes, we saw a 0.64 (95% CI 0.51 to 0.91) decrease in the odds of developing stigmatizing attitudes, given all the other variables in the model being held constant. Under the same assumptions, for a 1-unit increase in gene–environment knowledge, we saw a 0.51 (95% CI 0.64 to 0.78) decrease in the odds of holding more stigmatizing attitudes.

**Table 5.** Estimation results for logistic regression

							95% CI for Exp (β)	
Variables	β	SE	Wald	df	p-Value	Exp (β)	Lower	Upper
Friendship with affected individuals	−1.360	0.552	6.11	1	NS	0.256	0.087	0.754
Female gender	0.592	0.248	5.71	1	<0.001	1.808	1.112	2.938
Gene–environment knowledge	−0.380	0.147	6.65	1	<0.001	0.684	0.512	0.913
Rejecting infectious causes	−0.342	0.052	44.10	1	<0.001	0.710	0.642	0.768
Constant	2.068	0.215	92.546	1	<0.001	7.912		

Model χ^2^=386.88, p<0.05.

df: degrees of freedom; NS: not statistically significant; SE: standard error.

Accuracy of gene–environment knowledge and rejection of infectious causes were associated with a reduction of stigmatizing attitudes against patients with podoconiosis. Table 6 shows that the probability of holding stigmatizing attitudes was predicted to be 83% for youth who endorsed all infectious causes, given that all the predictors are set to their mean values. The probability for youth who endorsed half (5) of the infectious causes decreased to 50%. Likewise, the probability of holding stigmatizing attitudes was predicted to be 70% for youth who scored 0 for gene–environment knowledge, decreasing to 41% for those scoring 4 on this scale.

**Table 6.** Predicted probabilities for youth having stigmatizing attitudes toward patients with podoconiosis

					95% CI	
Delta margin	Margin	SE	Z	p>Z	Lower	Upper
Rejection of infectious causes at 0	0.8342	0.0350	23.79	<0.001	0.7655	0.9029
Rejection of infectious causes at 5	0.5041	0.0289	17.39	<0.001	0.4473	0.5618
Rejection of infectious causes at 10	0.1668	0.0402	4.15	<0.001	0.088	0.2456
Gene–environment accuracy at 0	0.6957	0.0618	11.25	<0.001	0.5745	0.8169
Gene–environment accuracy at 2	0.5567	0.2586	20.95	<0.001	0.5046	0.6088
Gene–environment accuracy at 4	0.4066	0.0509	7.98	<0.001	0.3067	0.5065

SE: standard error.

## Discussion

The findings of this report show that many youth in southern Ethiopia still have a desire to social distance from patients with podoconiosis, showing the lingering stigmatizing attitudes in podoconiosis-endemic communities. A number of prior studies have reported that stigmatization of those affected by podoconiosis is widespread among Ethiopian adults.^16–19^ Although stigmatizing attitudes and discrimination are not the same, stigmatizing attitudes can be strong indicators of discrimination against patients^33^ and can lead to a lack of support or empathy for affected people, leaving patients marginalized.

There have been few studies on the risk factors that account for stigmatizing attitudes among unaffected people in podoconiosis-endemic communities. We found a statistically significant association between stigmatizing attitudes towards patients with podoconiosis and factors such as gender, friendship with affected individuals, accuracy of gene–environment knowledge and rejection of infectious causes. Females were more likely to develop stigmatizing attitudes towards patients with podoconiosis. The tendency for females to have more stigmatizing attitudes towards patients is not well established in the literature. In a number of HIV/AIDS-related stigma studies, gender has not been reported to be an important factor in influencing stigmatizing attitudes towards PLWHA.^24,34,35^ Thus the influence of gender on stigmatizing attitudes, as noted in the present study, merits further investigation.

Friendship with an affected person was associated with less stigmatizing attitudes towards patients. One explanation could be that in-person contact with patients enhanced rural youth's understanding of the causes of podoconiosis, which was among the predictors of less stigmatizing attitudes. This is supported by our prior report from the same research project showing that friendship with patients helped rural youth to develop accurate knowledge and dispelled misconceptions about the causes of podoconiosis.^29^ Furthermore, the possible intimate and enduring personal relationships in friendship circles may have helped youth to develop a more humane and positive attitude towards patients. This finding is consistent with a prior study on HIV-related stigma, where familiarity with PLWHA was associated with less stigmatizing attitudes.^25^ Across a wide range of stigmatizing conditions, unaffected people have few meaningful relationships with affected individuals. Lack of contact with patients may foster discomfort, distrust and fear.^36^ The fact that personal contact with patients helps to reduce stigmatizing attitudes may indicate the possibility of recruiting and utilizing podoconiosis-affected individuals in stigma reduction interventions. Facilitating contact between persons affected by a particular condition and the general community has been reported to be effective in improving negative attitudes.^33^ A systematic review of anti-stigma programmes that have targeted college students found that in-person contact with patients was among the most viable types of intervention to change stigmatizing attitudes and reduce social distance.^37^ A recent study found that African American cancer survivors have high knowledge about breast and cervical cancer screening and the capacity to provide narratives about their experiences that the target population consider reliable.^38^

In the present study, more accurate beliefs about the cause of disease were associated with youth endorsing less stigmatizing attitudes. The marked relationship between disease knowledge and stigmatizing attitudes has been documented for other health conditions. HIV and related studies conducted in China,^23^ Korea^35^ and Nigeria^24^ have shown that accurate understanding predicts less stigmatizing attitudes towards patients. Similarly, enhancing people's understanding about the causes of disease has been reported to reduce stigma related to health conditions such as mental illness,^39^ epilepsy^40^ and albinism. Our findings showed that the accuracy of gene–environment knowledge was positively correlated with less stigmatizing attitudes towards those affected by podoconiosis. A prior study indicated that expanding public understanding of gene–environment influences on health could reduce health disparities and stigma directed towards patients.^41^ Investigating public understanding and beliefs about the contribution of gene–environment interaction to the development of health conditions and its association with stigmatizing attitudes is a relatively new area of study in Ethiopia.

Studies conducted in southern Ethiopia have reported that beliefs about infectious causes fuelled stigmatizing beliefs.^18,19^ In this study, rejecting infectious causes of podoconiosis was also associated with less stigmatizing attitudes, suggesting that dispelling beliefs related to podoconiosis being contagious could be one of the best ways to reduce stigmatizing attitudes. Nevertheless, it should be noted that it is difficult to change beliefs in infectious causes.^42^ Attempts to enhance public understanding of diseases should consider the local viewpoint, language and specific fears and beliefs.^43^ Information-based interventions that fill the knowledge gap about health conditions and dispel myths are among the most common approaches when addressing public stigma.^6^ Interaction with HEWs has been shown to be associated with greater gene–environment knowledge and reduced beliefs in infectious causes.^29^ Overall, our data appear to support the notion that improving the accurate understanding of disease could be highly effective against stigmatizing attitudes.

Our results did not show any significant association between formal education and stigmatizing attitudes towards patients with podoconiosis. Yet it ought to be noted that most of the youth did not have exposure to higher education (i.e. college or university) that could have expanded their access to general health information and humane approaches to people. Research has long attempted to clarify the relationship between education and stigmatizing attitudes towards patients. Several studies have reported that a higher level of education is a significant determinant of less stigmatizing attitudes towards PLWHA.^23,24^ A recent study further showed that unfavourable attitudes towards persons with albinism were associated with illiteracy.^44^ The implication being that higher levels of education exposes one to more accurate knowledge of causes of health conditions that may lessen negative attitudes. Indeed, in our previous report regarding rural youth's understanding of the gene–environment contribution to podoconiosis, we found that youth with a formal education had more accurate knowledge about causes of podoconiosis.^29^ However, a higher level of education did not have a direct effect on reported stigma beyond accurate knowledge. Thus rural youth across different education levels might benefit from educational programmes designed specifically to reduce their stigmatizing attitudes towards individuals affected by podoconiosis.

The data presented have several strengths. We employed a reliable scale to assess stigmatizing attitudes that has been used in prior studies of podoconiosis. Similarly, validated scales for gene–environment knowledge and beliefs in infectious causes were used. In addition, the study had a very high participation rate.

There were also some limitations to the data. The survey was conducted in only two rural communities and the findings may not be generalizable to other rural areas with diverse geographic and socio-economic conditions. It was not possible to rule out social desirability bias of participants’ reports. If there was intentional underreporting of stigmatizing attitudes, the data may underestimate these. Additionally, we only measured attitudes of rural youth and this may not be a sufficient indicator of acts of discrimination against patients. Assessing discriminatory acts against patients with podoconiosis and the determinants of these acts, using both quantitative and qualitative methods, may help identify ways to reduce the stigma against patients.

## Conclusions

Like other NTDs, podoconiosis elicits stigmatizing attitudes that could translate into discriminatory acts towards patients. To date, podoconiosis stigma research has focused primarily on the experiences of those affected by the condition and the attitudes of unaffected adults rather than attitudes of the unaffected population and youth. Our study explored public stigmatizing attitudes among rural youth in Ethiopia and potential risk factors. Stigma reduction interventions should aim to include the wider community to bring about attitudinal changes and foster understanding and acceptance, particularly among groups who lack accurate knowledge about podoconiosis. A previous intervention trial in southern Ethiopia indicated that enhancing gene–environment understanding could be a potentially powerful approach to reduce endorsed stigma towards affected individuals. The intervention indicated that culturally appropriate gene–environment education, i.e. the sun sensitivity metaphor, could help explain how individuals may inherit ‘soil sensitivity traits’, not the disease itself. On top of encouraging preventive actions, such strategies can minimize deterministic attitudes and improve negative attitudes towards patients.^20^ It has been suggested that public stigma reduction strategies will be most impactful when they enhance public awareness via information-based interventions and facilitate contact between patients and the wider community to change negative stereotypes.^6,33^ Thus we call for health promotion strategies that utilize local resources to enhance community understanding of gene–environment influences and reduce beliefs about infectious causes. Employing affected individuals in such efforts may be of great benefit.

## Data Availability

All data and material are available from the corresponding author upon reasonable request.
